# TORC1 signaling inhibition by rapamycin and caffeine affect lifespan, global gene expression, and cell proliferation of fission yeast

**DOI:** 10.1111/acel.12080

**Published:** 2013-05-02

**Authors:** Charalampos Rallis, Sandra Codlin, Jürg Bähler

**Affiliations:** Department of Genetics, Evolution & Environment and Institute of Healthy Ageing, University College LondonGower Street – Darwin Building, London, WC1E 6BT, UK

**Keywords:** cell proliferation, chronological aging, gene regulation, *Schizosaccharomyces pombe*, Target of Rapamycin, translation

## Abstract

Target of rapamycin complex 1 (TORC1) is implicated in growth control and aging from yeast to humans. Fission yeast is emerging as a popular model organism to study TOR signaling, although rapamycin has been thought to not affect cell growth in this organism. Here, we analyzed the effects of rapamycin and caffeine, singly and combined, on multiple cellular processes in fission yeast. The two drugs led to diverse and specific phenotypes that depended on TORC1 inhibition, including prolonged chronological lifespan, inhibition of global translation, inhibition of cell growth and division, and reprograming of global gene expression mimicking nitrogen starvation. Rapamycin and caffeine differentially affected these various TORC1-dependent processes. Combined drug treatment augmented most phenotypes and effectively blocked cell growth. Rapamycin showed a much more subtle effect on global translation than did caffeine, while both drugs were effective in prolonging chronological lifespan. Rapamycin and caffeine did not affect the lifespan via the pH of the growth media. Rapamycin prolonged the lifespan of nongrowing cells only when applied during the growth phase but not when applied after cells had stopped proliferation. The doses of rapamycin and caffeine strongly correlated with growth inhibition and with lifespan extension. This comprehensive analysis will inform future studies into TORC1 function and cellular aging in fission yeast and beyond.

## Introduction

Budding yeast (*Saccharomyces cerevisiae*) has been instrumental in the elucidation of basic mechanisms of cellular aging. Chronological lifespan (CLS), defined as the time yeast cells survive in a nondividing state, helps to understand the postmitotic aging of somatic cells (Fontana *et al*., 2010; Kaeberlein, 2010). Fission yeast (*Schizosaccharomyces pombe*) is only distantly related to budding yeast and thus provides a valuable complementary model system. Conserved nutrient-signaling pathways, such as Target of Rapamycin (TOR), affect aging from yeast to mammals (Laplante & Sabatini, 2012).

Target of Rapamycin proteins are serine/threonine kinases that control eukaryotic cell growth in response to nutrients (Wullschleger *et al*., 2006). In mammalian cells, a single TOR kinase is associated with two TOR-containing complexes, TORC1 and TORC2. Fission yeast contains two TOR kinases, Tor1p and Tor2p. Tor2p is essential and is mainly associated with TORC1 which positively regulates protein synthesis, metabolism, and transcription (Alvarez & Moreno, 2006; Uritani *et al*., 2006). Tor1p is associated with TORC2 which is implicated in DNA damage, telomere length, gene silencing, and stress response (Weisman, 2010). TORC1 promotes vegetative cell proliferation and inhibits nitrogen-starvation responses such as sexual differentiation and amino acid uptake, while TORC2 has opposite roles in these processes (*et al*., Wullschleger 2006; Weisman, 2010; Ikai *et al*., 2011). Disruption of TORC1, or depletion of Tor2p kinase, results in phenotypes similar to that of wild-type cells deprived of nitrogen, suggesting that TORC1 regulates growth in response to nitrogen availability (Matsuo *et al*., 2007; Weisman, 2010).

In several organisms, TOR can be inhibited by rapamycin, a macrolide which initially gained attention owing to its broad, anti-proliferative properties. Rapamycin forms an intracellular complex with the isomerase FKBP12 that then binds to the TOR kinase (*et al*., Wullschleger 2006). Rapamycin shows a strong inhibitory effect on vegetative growth in budding yeast (Heitman *et al*., 1991; Koltin *et al*., 1991). Although fission yeast contains a functional ortholog of FKBP12, Fkh1p (Weisman *et al*., 2001; Ikai *et al*., 2011), the effects of rapamycin in this organism are somewhat confusing. It has been reported that rapamycin does not affect growth (Weisman, 2010), although recent data show that *S. pombe* TORC1 is sensitive to rapamycin (Takahara & Maeda, 2012), and high doses commit cells to mitotic entry (Petersen & Nurse, 2007).

A fundamental function of TORC1 signaling is to promote protein synthesis by regulating the phosphorylation of the eIF4E-binding protein (4E-BP1) and the ribosomal S6 kinases (Huang & Manning, 2008). TOR signaling, and particularly TORC1, also plays a pro-aging role in all organisms tested. In budding yeast, for example, deletion of TOR pathway genes or treatment with rapamycin prolongs the CLS (Powers *et al*., 2006). A role for TOR in lifespan has been confirmed in metazoa: worms, flies, and mice treated with rapamycin live longer than untreated controls (Laplante & Sabatini, 2012). The conservation of the TOR kinases and their role in aging suggests that rapamycin could represent the first drug that prolongs the lifespan in multiple species. In fission yeast, TOR signaling has not been linked to cellular aging, but *S. pombe* TORC1 controls the phosphorylation status of the S6 ribosomal subunits (Nakashima *et al*., 2010).

Target of Rapamycin proteins belong to the phosphatidylinositol kinase-related kinase family (PIKK). PIKKs can also be inhibited to varying degrees by caffeine that preferentially inhibits mammalian TOR *in vivo* (Sarkaria *et al*., 1999; Block *et al*., 2004), and it specifically targets TORC1 in budding yeast (Wanke *et al*., 2008). In fission yeast, caffeine sensitivity has been implicated in diverse cellular processes, including oxidative stress response, the calcineurin pathway, as well as cell morphology and chromatin remodeling (Calvo *et al*., 2009). Caffeine extends lifespan in budding yeast (Wanke *et al*., 2008) and worms (Lublin *et al*., 2011). Moreover, emerging evidence suggests that caffeine affects the healthspan of organisms and elicits broad beneficial effects. Caffeine elicits a neuroprotective function that might prevent age-related cognitive decline and neuron pathology in Parkinson’s disease (Rosso *et al*., 2008). Caffeine also prevents age-associated recognition memory decline in old mice (Costa *et al*., 2008) and ameliorates pathological traits that are observed in a transgenic model of proteotoxicity associated with Alzheimer’s disease (Lublin *et al*., 2011).

Here, we report specific effects of rapamycin and caffeine, singly and combined, on CLS, gene expression signatures, protein translation, and cell proliferation in fission yeast. The two drugs differentially affect the different cellular processes in a TORC1-dependent manner, and combined drug treatment often enhances the resulting phenotypes. These findings afford insight into TORC1 function and cellular aging, and they provide a basis to further analyze these fundamental processes.

## Results

### Rapamycin and caffeine prolong chronological lifespan by TORC1 inhibition

**Figure 1:**
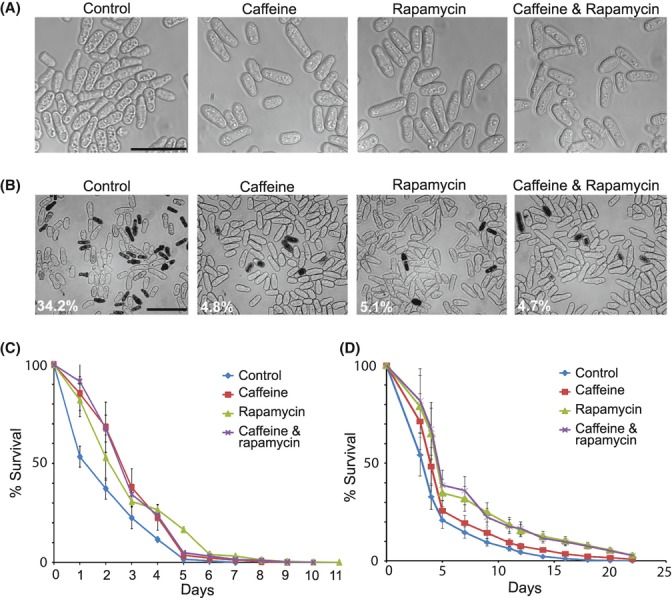
Rapamycin and caffeine reduce cellular aging and increase lifespan. (A) Fission yeast cells after 4 days in stationary phase in YES with different drug treatments as indicated. Bar equals 20 μm. (B) Phloxin B staining of cells after 2 days in stationary phase in YES with the different drug treatments. Percentages of red-stained (dead) cells are indicated at bottom left. Bar equals 30 μm.(C) Survival curves of wild-type cells in YES media with or without drug treatment as indicated.(D) Survival curves of wild-type cells in EMM media with or without drug treatment as indicated. Caffeine and rapamycin are used at concentrations of 10 mm and 100 μg mL^−1^, respectively.

Given the effects of TOR inhibition on lifespan in other organisms, we examined whether rapamycin and caffeine affect cellular aging in *S. pombe*. We also combined caffeine and rapamycin to maximize TORC1 inhibition (Takahara & Maeda, 2012). Old cells in stationary phase show an overall shrunken phenotype and oversized vacuoles (Roux *et al*., 2009). Compared to untreated control cells, these aging phenotypes appeared much less pronounced in cells treated with caffeine, rapamycin, or a combination of the two drugs (Fig. 1A). In addition, the drug-treated cells exhibited a lower proportion of phloxin B staining than control cells, indicating decreased cell death (Fig. 1B). Thus, rapamycin and caffeine seem to delay the aging and death of fission yeast cells. Accordingly, the drug-treated cells in rich media also showed substantially prolonged CLS in both rich medium (Figs 1C, S1A) and minimal medium (Figs 1D, S1B). In rich medium, treatment with rapamycin alone did lead to slightly longer maximal lifespan (Fig. S1A), but not median lifespan (Fig. 1C), than did combined drug treatment, although no such effect was observed in minimal medium (Figs 1D, S1B). In any case, rapamycin led to a stronger maximal CLS extension than did caffeine.

Acidification of the media due to the production of acetic acid has been shown to play a role in limiting the CLS of budding yeast (Murakami *et al*., 2011). Although acetic acid in fission yeast stationary phase cultures is believed to not affect cell survival (Zuin *et al*., 2010), caffeine and rapamycin might influence the pH of the media or enhance the resistance to acidification. We therefore measured, in parallel, cell growth and pH in media with and without the addition of caffeine or rapamycin. The drug-treated cell cultures showed similar patterns of acidification as nontreated cultures, with the pH dropping to around 5.8–6.0 during entry into stationary phase (Fig. S2A,B). In addition, we assessed the CLS in phosphate-citrate-buffered medium at both pH 6.0 and pH 4.0 (Fig. S2C,D). This analysis showed that the CLS was not affected by the different pH values in the buffered media. Moreover, caffeine and rapamycin did extend the CLS in both buffered media as it did in the nonbuffered media (Fig. S2C,D). We therefore conclude that caffeine and rapamycin do not affect the CLS via the pH of the media under the conditions analyzed.

**Figure 2:**
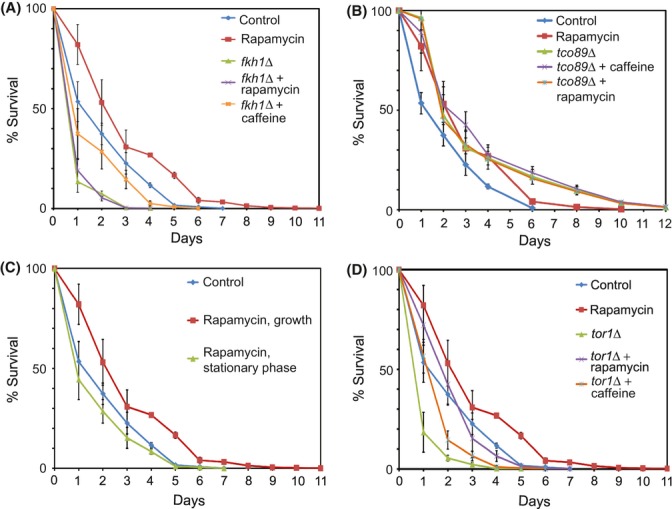
Lifespan extension via caffeine and rapamycin is TORC1-dependent. All CLS assays were performed in YES media at concentrations of 10 mm and 100 μg mL^−1^ of caffeine and rapamycin, respectively. (A) Survival curves of wild-type and *fkh1Δ* cells with or without rapamycin treatment as indicated. (B) Survival curves of wild-type and *tco89Δ* cells with or without rapamycin treatment as indicated. (C) Survival curves of wild-type cells with or without rapamycin treatment during growth or stationary phase as indicated. (D) Survival curves of wild-type and *tor1Δ* cells media with or without rapamycin treatment as indicated.

Rapamycin has been reported to interfere with TOR signaling by binding to the FKBP12 isomerase, called Fkh1p in *S. pombe* (Weisman *et al*., 2001; Weisman, 2010; Ikai *et al*., 2011). Notably, unlike caffeine, rapamycin did not extend the CLS in a *fkh1* deletion mutant (*fkh1Δ*) (Fig. 2A). This result suggests that rapamycin, but not caffeine, acts through Fkh1p to inhibit TOR signaling and extend lifespan. However, this finding was somewhat confounded as *fkh1Δ* cells were themselves more short-lived than wild-type cells (Fig. 2A), probably due to the lack of Fkh1p isomerase function that is independent of TOR signaling. To further test whether rapamycin and caffeine extend lifespan through TORC1 inhibition, we examined the CLS of *tco89Δ* mutant cells that lack a nonessential core component of TORC1 (Ikai *et al*., 2011). The *tco89Δ* cells were long-lived compared to wild-type cells, and their CLS was not further extended by rapamycin or caffeine treatment (Fig. 2B). These data further suggest that interference with TORC1 signaling leads to extended lifespan and that rapamycin, and probably also caffeine, affects the CLS by targeting TORC1. Accordingly, rapamycin extended CLS only when applied to growing cells, when TORC1 is active, and it did not affect lifespan when applied after cells had stopped to grow and entered stationary phase (Fig. 2C). This result shows that rapamycin acts during cell proliferation to subsequently prolong lifespan in nongrowing cells.

To check whether TORC2 might also affect CLS, independently of TORC1, we examined the lifespan of a *tor1Δ* mutant lacking the Tor1p component of TORC2 (Hartmuth & Petersen, 2009; Ikai *et al*., 2011). The *tor1Δ* cells showed a shortened CLS similar to *fkh1Δ* cells (Fig. 2D). Unlike *fkh1Δ* and *tco89Δ* cells, however, *tor1Δ* cells showed an extended lifespan after treatment with either rapamycin or caffeine, resulting in a CLS similar to wild-type cells (Fig. 2D). Thus, the drugs do not target TORC2 to increase the CLS. Taken together, these data indicate that TORC1 and TORC2 play antagonistic roles in shortening and extending the CLS, respectively, and that the lifespan extension of nongrowing cells by rapamycin and caffeine is mediated through inhibition of TORC1 signaling while the cells are still growing.

### Rapamycin and caffeine inhibit global translation mediated by S6 kinase

**Figure 3:**
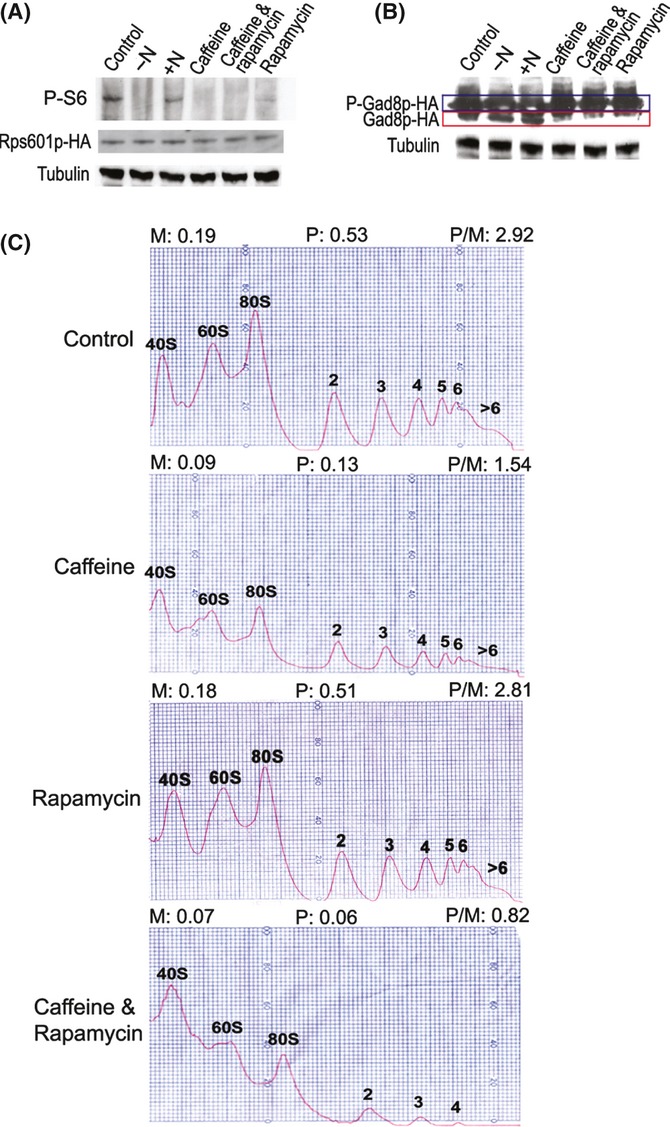
Effects of rapamycin and caffeine on TORC1-mediated translational control. (A) Western blot showing phosphorylation status of S6 proteins, using anti-PAS antibody, for cells grown in minimal medium supplemented with nitrogen (control), 1 h after removing nitrogen (−N), 30 min following nitrogen refeeding (+N), or with different drug treatments as indicated. Rps601p-HA and α-tubulin were used as loading controls. Caffeine and rapamycin are used at concentrations of 10 mm and 100 μg mL^−1^, respectively. (B) Western blot showing phosphorylation status of Gad8p-HA, with conditions as in (A). Phosphorylated and nonphosphorylated Gad8p are highlighted in blue and red boxes, respectively. (C) Polysome profiles from cells treated for 30 min in the absence (control) or presence of different drugs as indicated at left. Monosomal (M) and polysomal (P) peak areas as well as polysome-to-monosome ratios (P/M) are indicated on top for each condition.

The TORC1 pathway promotes protein translation, which is mediated in part via phosphorylation of ribosomal S6 proteins by S6 kinases (Urban *et al*., 2007; Wei *et al*., 2008; Nakashima *et al*., 2010). In *S. pombe,* candidates for such S6 kinases are Sck1p, Sck2p, Gad8p and Psk1p and two ribosomal S6 proteins have been identified, Rps601p and Rps602p (Nakashima *et al*., 2010). To test whether the effects of rapamycin and caffeine on cellular aging are mediated via S6 kinases, we used an anti-PAS [Phospho (S/T)-Akt substrate] antibody that detects the phosphorylated forms of S6 proteins. Cells treated with rapamycin, caffeine, or a combination of both drugs showed substantially decreased S6 protein phosphorylation, similar to nitrogen-starved cells (Fig. 3A). Caffeine, alone or combined with rapamycin, resulted in the strongest depletion of S6 protein phosphorylation, while partial phosphorylation was maintained in rapamycin-treated cells. We conclude that caffeine and rapamycin inhibit the TORC1-regulated phosphorylation by S6 kinases.

We tested whether rapamycin and caffeine also affect TORC2 function, which phosphorylates the S6 kinase-related Gad8p (Ikeda *et al*., 2008). We did not detect any decrease in either the phosphorylation or the protein levels of Gad8p in drug-treated cells compared to control cells; on the contrary, drug treatment resulted in a slight decrease in nonphosphorylated Gad8p (Fig. 3B). As a control, nitrogen availability did not affect the phosphorylation status or protein levels of Gad8p (Fig. 3B), as expected because TORC2 signaling is not affected by nitrogen (Nakashima *et al*., 2010; Ikai *et al*., 2011). Thus, while TORC1 function is inhibited by rapamycin and caffeine, TORC2 seems to become more active by the drug action. This result is consistent with the lifespan data (Fig. 2) and with the antagonistic roles played by the two TOR complexes in *S. pombe* (Weisman, 2010).

To test whether the inhibition of S6 protein phosphorylation by rapamycin and caffeine affects protein translation, we analyzed polysome profiles. Monosome and polysome peaks as well as polysome-to-monosome (P/M) ratios were determined in drug-treated and control cells as an estimate for global translational activity (Fig. 3C). Compared to untreated control cells, the P/M ratio decreased ∼2-fold in caffeine-treated cells and ∼3.6-fold in cells treated with both drugs, while rapamycin alone showed only a subtle effect (Fig. 3C). The mild effect of rapamycin on global translation is consistent with the finding that some S6 protein phosphorylation remained after rapamycin treatment (Fig. 3A). Taken together, we conclude that caffeine leads to a global reduction in protein translation by inhibition of TORC1-mediated S6 protein phosphorylation, and that this inhibition is augmented when combined with rapamycin.

### Rapamycin and caffeine trigger gene expression reprograming similar to nitrogen deprivation and tor2 mutant

**Figure 4:**
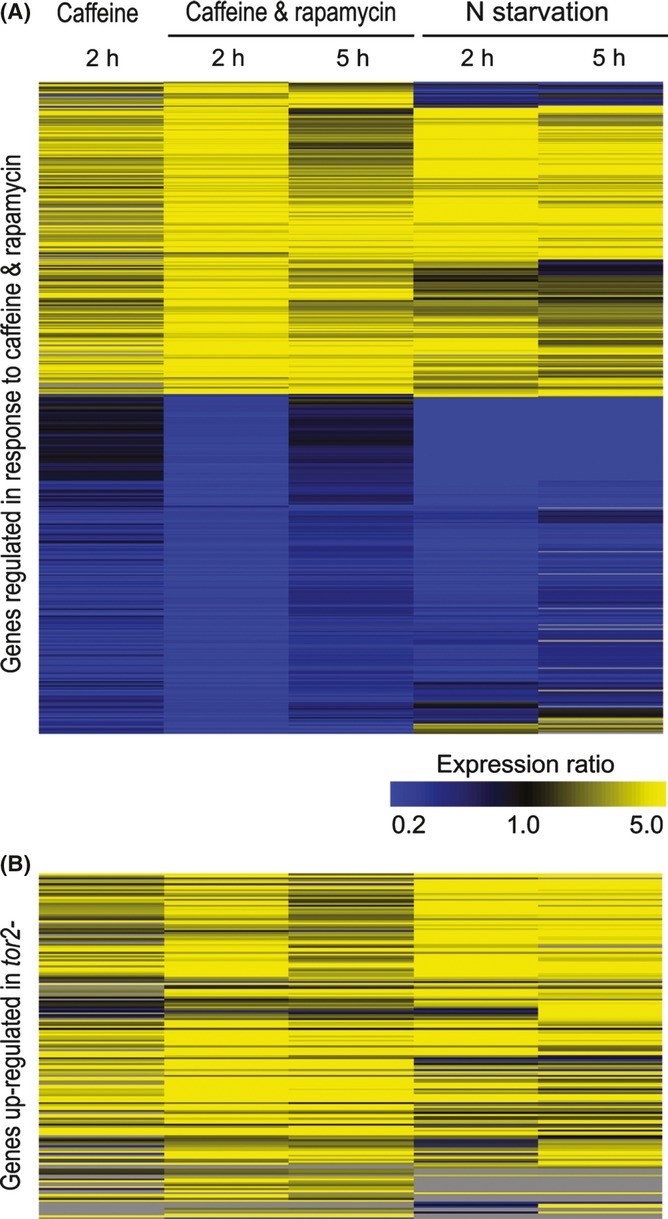
Caffeine triggers gene expression reprograming like nitrogen deprivation and TORC1 inhibition. (A) Hierarchical clustering analysis with columns representing experimental timepoints and rows representing 539 genes whose mRNA levels changed >3-fold after exposure to 10 mm caffeine and 100 μg mL^−1^ rapamycin in at least 1 timepoint, and which were measurable in at least three of the four conditions shown. Columns 1–3: mRNA expression in cells treated with caffeine singly or combined with rapamycin after 2-h and 5-h treatment as indicated, relative to expression in the same cells before treatment (this study). Columns 4 and 5: mRNA expression after 2 and 5 h of nitrogen starvation as indicated, relative to expression in the same cells before starvation. Relative expression levels are color coded as indicated at bottom, with missing data in gray. (B) Cluster analysis as in (A), showing the expression in caffeine singly or combined with rapamycin and nitrogen starvation for the 177 genes whose mRNA levels were induced in *tor2* mutants (Matsuo *et al*., 2007) and produced expression data in at least 2 of the 4 conditions shown.

To further analyze the effects of rapamycin and caffeine treatment in *S. pombe*, we performed gene expression profiling using DNA microarrays. Rapamycin treatment only led to subtle effects on gene expression, unless a poor nitrogen source was used (data not shown). Caffeine, however, triggered a large expression response, including hundreds of up- and down-regulated genes (Fig. 4A). This response was further enhanced by combined treatment with caffeine and rapamycin. Notably, the gene expression signature of drug-treated cells was highly similar to the signature of cells deprived of nitrogen (Fig. 4A) (Mata & Bähler, 2006). Accordingly, the drugs led to >3-fold down-regulation of 350 genes for ribosomal proteins and other translation- and growth-related proteins, and >3-fold up-regulation of 343 genes involved in meiotic differentiation (Mata *et al*., 2002) and core environmental stress response (Chen *et al*., 2003), including autophagy, membrane transport, and amino acid permease genes (Table S1). Only 20 genes that were up-regulated during drug treatment were down-regulated during nitrogen depletion, including genes involved in amino acid metabolism, transporters, and noncoding RNAs (Fig. 4A, top; Table S1).

Nitrogen is a major cue for proliferation of *S. pombe* cells, and the TORC1 pathway controls gene expression in response to nitrogen and amino acids (Yanagida, 2009), while inhibition of TORC1 leads to similar phenotypes as those triggered by nitrogen deprivation and promotes sexual differentiation (Uritani *et al*., 2006; Matsuo *et al*., 2007). We therefore expected that caffeine leads to gene expression changes as in cells defective for TORC1 signaling. Indeed, we found that genes reported to be up-regulated in a *tor2* mutant (Matsuo *et al*., 2007) were also up-regulated in cells treated with caffeine, either singly or combined with rapamycin (Fig. 4B). Thus, treatment with caffeine mimics nitrogen deprivation and TORC1 inhibition at the level of transcriptome regulation.

Upon nitrogen removal, *S. pombe* cells stop growth and arrest in G1 phase as quiescent cells, which become stubby and shrink in volume ∼2-fold (Yanagida, 2009). Inhibition of Tor2p function mimics all of these phenotypes (Uritani *et al*., 2006; Matsuo *et al*., 2007). We therefore examined whether the two drugs also lead to phenotypes associated with nitrogen deprivation. Addition of caffeine and rapamycin led to some decrease in cell size, but the drug-treated cells were not as stubby as nitrogen-starved cells (Fig. S3). Moreover, the drug-treated cells became arrested in G2 phase of the cell cycle, not in G1 phase like nitrogen-deprived cells (Fig. S3 and data not shown). As previously described (Wang *et al*., 1999), our data also revealed that some caffeine-treated cells transiently delayed cell division and became multiseptated (Fig. S3). We conclude that despite triggering gene expression responses similar to nitrogen-deprived and *tor2* mutant cells, treatment with rapamycin and caffeine does not mimic the quiescent state with respect to cell morphology and G1 cell-cycle arrest.

### Rapamycin and caffeine differentially inhibit cell growth and division via TORC1

**Figure 5:**
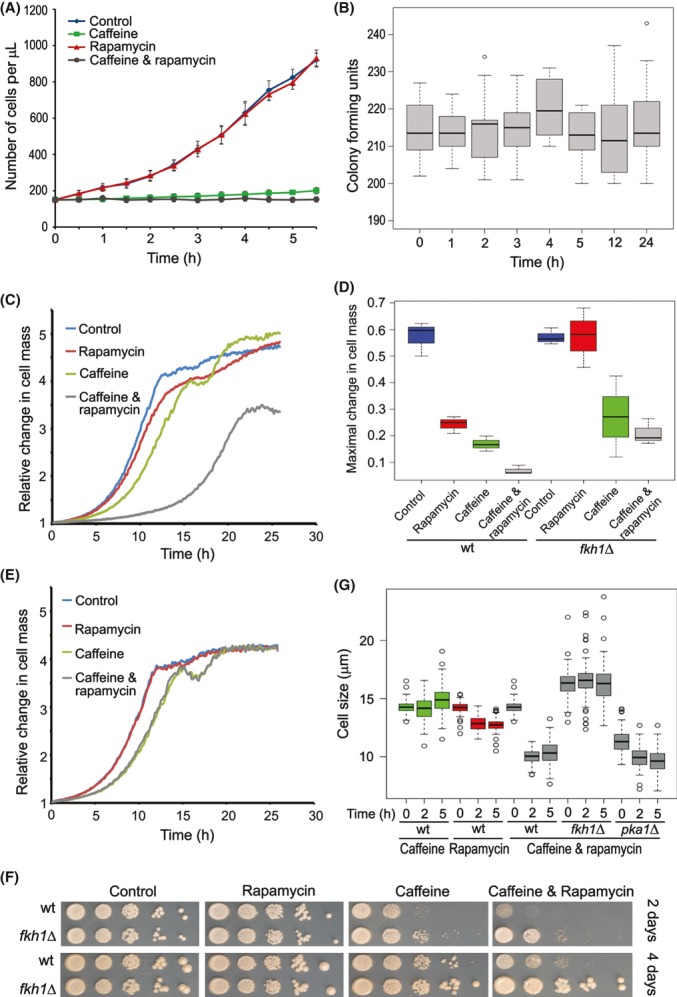
Rapamycin and caffeine inhibit cell growth and division. All these assays were performed in YES media, at concentrations of 10 mm and 100 μg mL^−1^ of caffeine and rapamycin, respectively. (A) Proliferation of cells in liquid medium without (control) or with different drug treatments as indicated. Error bars represent standard deviations of 4 measurements. (B) Cell viability (colony forming units) during a 24-h treatment with both rapamycin and caffeine. Box plots were generated using 10 measurements for each timepoint. (C) Growth curves of wild-type cells in liquid medium without (control) or with different drug treatments as indicated. (D) Maximal growth slopes (relative biomass increase per min) for wild-type and *fkh1Δ* cells without (control) or with different drug treatments as indicated. Five repeats were conducted for each condition. (E) Growth curves of *fkh1Δ* cells in liquid YES medium without (control) or with different drug treatments as indicated. (F) Serial dilution of wild-type and *fkh1Δ* cells were spotted on agar plates without (control) or with different drug treatments as indicated. Cell colonies are shown after 2 (top) and 4 (bottom) days of incubation. (G) Cell size at division following rapamycin, caffeine, or combined drug treatment in wild-type (wt) and *fkh1Δ* and *pka1Δ* mutant cells as indicated.

In stark contrast to other organisms, rapamycin does not seem to arrest cell growth in *S. pombe* (Weisman, 2010). Given the effects of rapamycin and caffeine on other TORC1-dependent processes (Figs 4), we re-examined the proliferation of cells treated with the two drugs. Cells treated with rapamycin showed a similar increase in cell numbers as untreated control cells, while cells treated with caffeine showed a much slower increase (Fig. 5A). The latter finding is consistent with the growth inhibition described at high doses of caffeine (Calvo *et al*., 2009). The combined treatment with rapamycin and caffeine seemed to further inhibit cell proliferation (Fig. 5A).

The maximal cell densities reached after extended incubation were also differentially affected by the drugs: after 16 h incubation, cells treated with rapamycin reached the same overall density as untreated control cells (1.6 × 10^8^ cells per mL), cells treated with caffeine grew to a density of only 8.7 × 10^6^ per mL, while cells treated with both drugs failed to proliferate altogether (1.6 × 10^5^ cells per mL). These effects on cell proliferation largely mirror the differential effects of the drugs on global translation (Fig. 3C). The number of viable cells did not decrease, even during extended treatment with both drugs (Fig. 5B). This finding indicates that caffeine and rapamycin lead to a reversible growth arrest and does not trigger cell death.

The cell density impacted the growth kinetics: cells incubated at high density recovered rapidly from drug treatment and started to grow normally (data not shown), possibly reflecting effective drug degradation by cellular metabolism. Moreover, some genetic strain backgrounds showed different kinetics of growth inhibition (data not shown). To further examine the effect of caffeine and rapamycin on the growth of *S. pombe* cells, we analyzed the growth dynamics of rapidly proliferating cell cultures for 24 h in the presence or absence of rapamycin and caffeine using a microculture system. Both rapamycin and caffeine led to delayed growth and decreased maximal growth rate, and combination of the two drugs markedly increased these effects (Fig. 5C,D). Although rapamycin alone did not affect the increase in cell numbers and thus cell-cycle duration (generation time), within a 5-h window (Fig. 5A), it did inhibit cell growth (Fig. 5C,D). This discrepancy may be explained by the smaller size of cells treated with rapamycin (Fig. 5G): these cells grow more slowly while the duration of the cell cycle is not altered, resulting in smaller cell size at division.

To investigate the mechanism of drug action on TORC1, we analyzed the effects of rapamycin and caffeine on growth in the absence of Fkh1p. In *fkh1Δ* cells, rapamycin had no effect on cell growth (Fig. 5D,E). In contrast, treatment of caffeine, alone or combined with rapamycin, inhibited cell growth even in *fkh1Δ* cells (Fig. 5D,E). These data support the view that rapamycin affects cell growth by inhibiting TORC1 via Fkh1p, while caffeine affects growth by inhibiting TORC1 independently of Fkh1p and/or by acting on other cellular processes. Qualitatively, the same findings were obtained when using minimal instead of rich medium (Fig. S4).

The growth inhibition by rapamycin was less evident on solid media, but caffeine and, even more so, the combined drugs greatly inhibited colony growth (Fig. 5F). This inhibition was partly dependent on Fkh1p, even for caffeine (Fig. 5F). These differences to the liquid media experiments might reflect the distinct timelines (days *vs* hours) and different physiological conditions in solid *vs* liquid cultures.

Cell size is tuned to the availability of different nutrients (Turner *et al*., 2012), and TOR signaling is involved in the nutrient-dependent coordination between cell growth and division in *S. pombe* (Ikai *et al*., 2011). We found that cell size at division was somewhat decreased following rapamycin treatment, and dramatically decreased after combined drug treatment, but not after treatment with caffeine alone (Fig. 5G). Notably, *fkh1Δ* cells were somewhat larger than wild-type cells, and they showed no cell size reduction in response to the combined drug treatment (Fig. 5G). This finding is consistent with data showing that rapamycin affects cell size via Fkh1p and TORC1 (Weisman, 2010; Ikai *et al*., 2011). As protein kinase A (PKA) signaling has also been implicated in cell size control (Turner *et al*., 2012), we examined whether rapamycin and caffeine might also target PKA. As expected, *pka1Δ* mutant cells were substantially smaller than wild-type cells, but the combined drug treatment led to an even further decrease to a final cell size similar to the minimum observed with drug-treated wild-type cells in the same treatment (Fig. 5G). We conclude that the combination of rapamycin and caffeine treatment affects cell size independently of PKA, by inhibiting TORC1 signaling.

Taken together, these data indicate that rapamycin and caffeine affect *S. pombe* cell growth and division to different degrees. Rapamycin slightly reduces cell growth in a TORC1-dependent manner but does not affect cell division, leading to smaller cells. Caffeine, on the other hand, reduces both cell growth and division, but cell size is not affected. Combined treatment of rapamycin and caffeine greatly inhibits cell growth, leading to highly decreased cell proliferation and cell size.

### Dosage effects of rapamycin and caffeine on cell growth and lifespan

**Figure 6:**
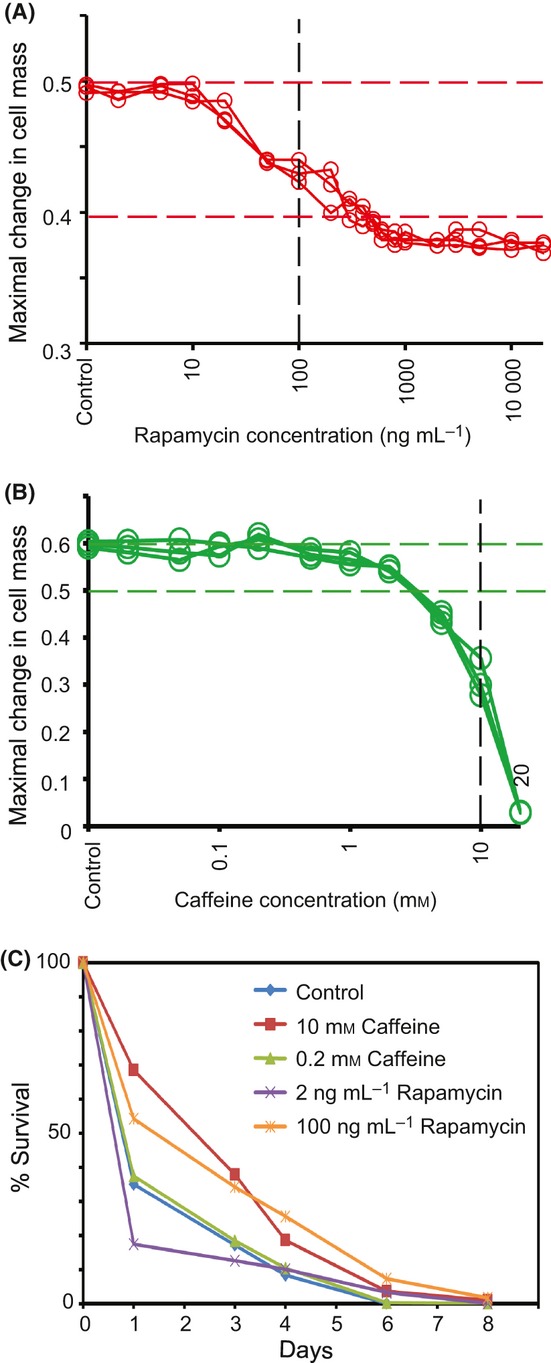
Rapamycin and caffeine doses correlate with growth inhibition and CLS extension. All these assays were performed in YES media. (A) Maximal growth slopes of cells treated with different concentrations of rapamycin as indicated. Vertical dashed lines indicate the standard concentrations of rapamycin used in the other assays of the paper. (B) Maximal growth slopes of cells treated with different concentrations of caffeine as indicated. Note the different scale of the *y*-axis compared to (A), indicated by the horizontal red and green dashed lines in (A) and (B), respectively. Vertical black dashed lines indicate the standard concentration of caffeine used in the other assays of the paper. (C) Survival curves of wild-type cells with or without different drug treatments as indicated.

To examine the relationships between the dose of rapamycin and caffeine and the resulting CLS extension and cell growth inhibition, we examined growth patterns and viability of cells treated with a range of concentrations of the two drugs. The maximal growth rate of the cells was decreased with increasing concentrations of both rapamycin and caffeine (Fig. 6A,B). While the rapamycin-dependent growth inhibition gradually and weakly increased with dose (Fig. 6A), the caffeine-dependent growth inhibition showed a more threshold-like effect with strong inhibition above ∼2 mm (Fig. 6B). Caffeine concentrations above 20 mm were lethal to fission yeast cells (data not shown).

Drug dosage also affected the CLS: cells treated with lower caffeine and rapamycin concentrations showed less pronounced increase in lifespan than those treated with higher concentrations (Fig. 6C and data not shown). We further analyzed the correlation between viability at different days of the CLS assay as a function of different doses of rapamycin and caffeine. Increases in both the rapamycin and caffeine concentrations, especially at the lower end of the doses tested, showed substantial positive effects on cell viability, while a saturation effect became evident at higher doses (Fig. S5). As caffeine was lethal for exponentially growing cultures at concentrations over 20 mm, it was not possible to determine CLS above this concentration. The growth inhibitory effect of rapamycin became saturated at concentrations above ∼500 ng mL^−1^, which is the same concentration resulting in maximal CLS extension. We conclude that increased concentrations of rapamycin and caffeine are strongly correlated both with increased growth inhibition and with lifespan extension.

## Discussion

This study presents a detailed analysis of rapamycin and caffeine effects on fission yeast cells, providing a framework to further study TOR function and cellular aging in this model organism. Rapamycin and caffeine led to multiple phenotypes via inhibition of TORC1 signaling: (i) increase in CLS; (ii) inhibition of global translation; (iii) reprograming of gene expression; and (iv) inhibition of cell proliferation. Rapamycin and caffeine differentially affected these conserved TORC1-dependent processes, and combination of the two drugs often augmented the phenotypes. The growth inhibition and lifespan extension depended on the dosage of both rapamycin and caffeine, with increased doses leading to increased phenotypic effects. In contrast to other organisms and as previously observed (Weisman, 2010), rapamycin did never lead to complete growth arrest in fission yeast cells, even at high doses. This surprising finding raises the possibility that rapamycin does not completely inhibit TORC1 or that TORC1 is dispensable for growth in fission yeast.

Notably, rapamycin did prolong the lifespan of nondividing cells only when applied while these cells were still growing and dividing but had no effect when applied after cells reached stationary phase. This finding could reflect that TORC1 is only active in growing cells, and reducing this activity ultimately extends the longevity of cells in stationary phase. This phenomenon is reminiscent of the finding that low glucose levels during cell growth promote extended lifespan of stationary phase cells via the PKA pathway (Roux *et al*., 2009). The nature of this intriguing information flow from growth to stationary phase is not clear, however. Rapamycin inhibited TORC1 activity via the FKBP12 isomerase ortholog Fkh1p. These findings establish for fission yeast a conserved action of rapamycin in TORC1 inhibition and longevity. Unlike TORC1 activity, which shortens the CLS, TORC2 activity seems to promote the CLS. Thus, TORC1 and TORC2 may play antagonistic roles with respect to longevity, as they do with respect to other cellular processes such as growth and meiotic differentiation (Weisman, 2010).

Unlike in budding yeast where acidification of growth media plays a role in limiting the CLS (Murakami *et al*., 2011), we found no evidence that pH changes are important in fission yeast for the effects on CLS reported here. Rapamycin and caffeine did not influence media acidification, and media buffered at two vastly different pH values had no effect on CLS in untreated cells or on its extension in drug-treated cells. These findings are consistent with results reported by Zuin *et al*. (2010) showing that acetic acid in fission yeast stationary cultures does not affect cell survival. Note that even in budding yeast, media acidification does not fully explain the role of TOR-S6K pathway on aging, because its inhibition still extends CLS in media that is not acidified and does not contain acetic acid (Wei *et al*., 2008).

Caffeine triggers the stress-activated protein kinase (SAPK) pathway in *S. pombe* (Zuin *et al*., 2010). The SAPK and TOR pathways may be antagonistically regulated (López-Maury *et al*., 2008), raising the possibility that inhibition of TORC1 signaling by caffeine causes the activation of SAPK. Indeed, caffeine treatment led to induction of stress response genes and repression of ribosomal and other growth-related genes while Sty1p was required for the caffeine-induced lifespan extension (Fig. 4). The gene expression changes observed could cause or support some of the drug-induced phenotypes such as growth and translational inhibition. We find that caffeine treatment in *sty1Δ* cells decreases rather than increases the CLS (unpublished data). These data suggest that Sty1p may be required for the CLS extension imposed by caffeine, as it is for the CLS extension by calorie restriction (Zuin *et al*., 2010). Despite triggering gene expression responses similar to nitrogen-deprived and *tor2* mutant cells, treatment with rapamycin and caffeine did not mimic the quiescent state with respect to cell morphology and G1 cell-cycle arrest (Yanagida, 2009). This finding suggests that reprograming of gene expression is not sufficient to trigger a quiescent state within the 5-h timeframe of our experiment.

Reduced protein translation has been linked to increased lifespan in several organisms (Harrison *et al*., 2009; Bjedov *et al*., 2010; Kaeberlein, 2010). The effects of rapamycin and caffeine on global translation are therefore consistent with the increase in CLS caused by the same drugs. As translation is required for cellular growth, it is also consistent with the two drugs inhibiting cell proliferation and with the correlation between slow growth and longevity as a function of drug concentrations (Figs 6; S5). However, rapamycin alone was sufficient for a substantial extension in lifespan (Fig. 2), while it had much more subtle effects on global translation and on phosphorylation levels of S6 proteins (Fig. 3). This apparent paradox suggests either that a weak inhibition of translation is more effective for longevity or that rapamycin can extend lifespan by mechanisms other than translation. One alternate mechanism could act via protection from DNA damage and genome instability in stationary phase; consistent with this idea, rapamycin has recently been shown to repress the phenotypes of *cut1* and *cut2* chromosome segregation mutants, which cause DNA damage (Ikai *et al*., 2011). In other organisms, rapamycin positively affects lifespan through elusive pathways apart from growth and translation (Wei *et al*., 2008; Harrison *et al*., 2009; Bjedov *et al*., 2010), possibly involving mitochondrial function (Pan & Shadel, 2009).

Fission yeast is bound to make unique contributions to defining both universal and specialized processes relevant to cellular aging and TOR signaling; its low-complexity genome, technical resources, and genetic tractability under tightly controlled conditions provide a potent platform, complementary to budding yeast, to identify relevant genetic factors involved in these processes. This study presents a basis for future mechanistic analyses of TORC1 control and CLS in fission yeast and will help identify new proteins functioning in these fundamental processes.

## Experimental procedures

### Strains and media

Wild-type cells were the 972 *h*^*−*^ strain. *S. pombe* deletion mutant strains, including *fkh1Δ, tco89Δ* and *tor1Δ* deletion mutants, were obtained from the Bioneer version 2.0 (Bioneer Corporation, Daejeon, Korea) library and PCR-verified for correct deletions at both junctions. The C-terminally HA-tagged *gad8* and *rps601* strains were generated as described (Bähler *et al*., 1998). Cell cultures were grown as indicated in yeast extract plus supplements (YES, 3% glucose) or EMM medium (2% glucose). Nitrogen deprivation experiments were performed in EMM without NH_4_Cl_2_. Cells were grown to an OD_600_ of 0.5, washed twice with EMM without NH_4_Cl_2_, and incubated for 1 h in EMM without NH_4_Cl_2_, when a sample was taken (−N). Cells were then centrifuged and resuspended in EMM with NH_4_Cl_2_ and incubated for 30 min, when another sample was taken (refeeding). Liquid cultures were grown at 32 °C with shaking at 130 rotations per minute.

### Caffeine and rapamycin sensitivity and stress assays by serial dilutions

*Schizosaccharomyces pombe* strains were rapidly grown in liquid YES cultures to an OD_600_ of 0.5. Ten-fold serial dilutions of cells were spotted, using replica platers for 48-well or 96-well plates (Sigma-Aldrich Ltd, Gillingham, UK), onto YES agar plates, with or without caffeine (10 mm) and/or rapamycin (100 ng mL^−1^). Plates were incubated at 32 °C.

### Measurement of cell size at division

Control and drug-treated cells were fixed in 4% formaldehyde for 10 min at room temperature, washed with 50 mm sodium citrate and 100 mm sodium phosphate, and stained with calcofluor (50 μg mL^−1^). Cells were photographed in a Zeiss microscope using the OpenLab program. At least 100 septated cells were counted and analyzed for each condition using the ImageJ package (Rasband, 1997–2007).

### Measurement of cell number during growth

Cell numbers were determined using a Beckman Coulter Counter system. Four independent dilutions of each culture were prepared and measured. For growth measurements in caffeine and rapamycin (Fig. 4), log-phase cultures in YES were separated into the following four samples: an untreated growth control, a sample supplemented with 10 mM caffeine (Calvo *et al*., 2009), a sample treated with 100 ng mL^−1^ rapamycin, and a sample treated with both caffeine and rapamycin at the doses above. We used 1.5 × 10^5^ cells to inoculate each culture.

### Growth assay

Growth curves under normal and various stress conditions were automatically determined by the BioLector microfermentation system (m2p-biolabs), using 48-well flowerplates, at 1.5 mL volume, 1000 rpm and 32 °C. The growth dynamics, maximum growth slopes, and maximum densities were calculated using the grofit R package (Kahm *et al*., 2010). For caffeine and rapamycin treatments, rapidly growing cultures at OD_600_ = 0.5 in YES were diluted at OD_600_ = 0.15 and were treated with the drugs at the concentrations indicated in the corresponding Fig. 5D,E. Same procedure was followed in Fig. S4, where EMM media were used instead of YES.

### Chronological lifespan assay

Cells were grown in YES or EMM as described (Roux *et al*., 2009). When cultures reached a stable maximal density, cells were harvested, serially diluted, and plated on YES plates. The measurement of colony-forming units (CFUs) was taken as timepoint 0 at the beginning of the CLS curve (i.e., 100% cell survival). Measurements of CFUs were conducted on the following days until cultures reached 0.1% of the initial cell survival. Error bars represent standard deviation calculated from three independent cultures, with each culture measured at least three times at each timepoint. To determine the CLS of cells treated with caffeine and/or rapamycin, cell cultures at OD_600_ = 0.15 were treated with the drugs at concentrations indicated in the corresponding figures. Survivals are indicated in Table S2. Survival curves were statistically analyzed with Kaplan–Meier survival plots and logrank tests using the survival R package (http://www.r-project.org/).

### Western blotting and antibodies

For protein preparations, cells were diluted in 6 mm Na_2_HPO_4_, 4 mm NaH_2_PO_4_.·H_2_O, 1% Nonidet P-40, 150 mm NaCl, 2 mm EDTA, 50 mm NaF supplemented with protease (PMSF) and phosphatase inhibitors (Sigma cocktails 1 and 2) together with glass beads. Cells were lysed in a Fastprep-24 machine. Anti-HA (H9658, Sigma) and anti-α-tubulin (B5-1-2, Sigma) were used at 1/5000, 1/5000 and 1/10,000 dilutions, respectively. Detection was performed using anti-mouse and anti-rabbit HRP-conjugated antibodies (1/5000 dilutions) with the ECL Western blotting detection system (GE Healthcare, Chalfont St Giles, UK) according to the manufacturer’s protocol.

### Polysome profiling

Translational profiles were acquired as previously described (Lackner *et al*., 2007). *S. pombe* cells were grown in liquid YES cultures to OD_600_ = 0.5. Caffeine and rapamycin (10 mm and 100 ng mL^−1^, respectively) were added alone and in combination. Cells were harvested after 30 min of drug treatment. *S. pombe* protein preparations were performed according to a standard protocol as described earlier (western blotting), but using a different lysis buffer (20 mm Tris–HCl pH 7.5, 50 mm KCL, 10 mm MgCl). Sucrose gradients (10–50%) were generated using a Biocomp Gradient Master, and protein preparations were loaded and centrifuged at 35 000 rpm for 2 h 40 min. Polysome gradients were then loaded to the fractionator to obtain the translational profiles.

### Expression microarrays

Cells were grown in EMM to OD_600_ = 0.5 and harvested. RNA was isolated followed by cDNA labeling (Lyne *et al*., 2003). For caffeine and rapamycin treatments (10 mm and 100 ng mL^−1^, respectively), rapidly growing cells (OD_600_ = 0.5) in EMM were treated with the drugs for 2 and/or 5 h as indicated in Fig. 4. For nitrogen starvation, rapidly growing cells (OD_600_ = 0.5) in EMM were washed twice with EMM without NH_4_Cl_2_ and incubated in this medium for 2 and 5 h before harvesting. Agilent 8 × 15K custom-made *S. pombe* expression microarrays were used, and hybridizations and subsequent washes performed according to the manufacturer’s protocols. The obtained data were scanned and extracted using GenePix, processed using R scripts for quality control and normalization (Lyne *et al*., 2003), and analyzed using GeneSpring GX3 (Agilent Technologies UK Ltd, Wokingham, UK). Two independent biological repeats with a dye swap were performed. Microarray data have been submitted to ArrayExpress (accession number E-MTAB-1489).

## Supporting Information

Fig S1 Survival assays of wild-type cells following drug treatment in YES and EMM media showing logarithmic scales of cell survival.

Fig S2 Parallel measurements of cell growth and pH in media without (control) or with drugs as indicated, and CLS in buffered YES media with or without drugs as indicated

Fig S3 Cell morphology and DNA content in caffeine and rapamycin-treated cells.

Fig S4 Growth analysis of cells in EMM media untreated (control) or treated with drugs as indicated.

Fig S5 Correlation of cell viability with the concentrations of rapamycin and caffeine administered to growing cells.

Table S1 List of genes regulated in cells treated with rapamycin and/or caffeine.

Table S2 Summary of all CLS data in this study.

## References

[b1] Alvarez B, Moreno S (2006). Fission yeast Tor2 promotes cell growth and represses cell differentiation. J. Cell Sci.

[b2] Bähler J, Wu JQ, Longtine MS, Shah NG, McKenzie A, Steever AB, Wach A, Philippsen P, Pringle JR (1998). Heterologous modules for efficient and versatile PCR-based gene targeting in *Schizosaccharomyces pombe*. Yeast.

[b3] Bjedov I, Toivonen JM, Kerr F, Slack C, Jacobson J, Foley A, Partridge L (2010). Mechanisms of life span extension by rapamycin in the fruit fly Drosophila melanogaster. Cell Metab.

[b4] Block WD, Merkle D, Meek K, Lees-Miller SP (2004). Selective inhibition of the DNA-dependent protein kinase (DNA-PK) by the radiosensitizing agent caffeine. Nucleic Acids Res.

[b5] Calvo IA, Gabrielli N, Iglesias-Baena I, Garcia-Santamarina S, Hoe KL, Kim DU, Sanso M, Zuin A, Perez P, Ayte J, Hidalgo E (2009). Genome-wide screen of genes required for caffeine tolerance in fission yeast. PLoS ONE.

[b6] Chen D, Toone WM, Mata J, Lyne R, Burns G, Kivinen K, Brazma A, Jones N, Bähler J (2003). Global transcriptional responses of fission yeast to environmental stress. Mol. Biol. Cell.

[b7] Costa MS, Botton PH, Mioranzza S, Souza DO, Porciuncula LO (2008). Caffeine prevents age-associated recognition memory decline and changes brain-derived neurotrophic factor and tirosine kinase receptor (TrkB) content in mice. Neuroscience.

[b8] Fontana L, Partridge L, Longo VD (2010). Extending healthy life span–from yeast to humans. Science.

[b9] Harrison DE, Strong R, Sharp ZD, Nelson JF, Astle CM, Flurkey K, Nadon NL, Wilkinson JE, Frenkel K, Carter CS, Pahor M, Javors MA, Fernandez E, Miller RA (2009). Rapamycin fed late in life extends lifespan in genetically heterogeneous mice. Nature.

[b10] Hartmuth S, Petersen J (2009). Fission yeast Tor1 functions as part of TORC1 to control mitotic entry through the stress MAPK pathway following nutrient stress. J. Cell Sci.

[b11] Heitman J, Movva NR, Hall MN (1991). Targets for cell cycle arrest by the immunosuppressant rapamycin in yeast. Science.

[b12] Huang J, Manning BD (2008). The TSC1-TSC2 complex: a molecular switchboard controlling cell growth. Biochem. J.

[b13] Ikai N, Nakazawa N, Hayashi T, Yanagida M (2011). The reverse, but coordinated, roles of Tor2 (TORC1) and Tor1 (TORC2) kinases for growth, cell cycle and separase-mediated mitosis in *Schizosaccharomyces pombe*. Open Biol.

[b14] Ikeda K, Morigasaki S, Tatebe H, Tamanoi F, Shiozaki K (2008). Fission yeast TOR complex 2 activates the AGC-family Gad8 kinase essential for stress resistance and cell cycle control. Cell Cycle.

[b15] Kaeberlein M (2010). Lessons on longevity from budding yeast. Nature.

[b16] Kahm M, Hasenbrink G, Lichtenberg-Fratte H, Ludwig J, Kchischo M (2010). Grofit:fitting biological growth curves with R. J. Stat. Software.

[b17] Koltin Y, Faucette L, Bergsma DJ, Levy MA, Cafferkey R, Koser PL, Johnson RK, Livi GP (1991). Rapamycin sensitivity in *Saccharomyces cerevisiae* is mediated by a peptidyl-prolyl cis-trans isomerase related to human FK506-binding protein. Mol. Cell. Biol.

[b18] Lackner DH, Beilharz TH, Marguerat S, Mata J, Watt S, Schubert F, Preiss T, Bähler J (2007). A network of multiple regulatory layers shapes gene expression in fission yeast. Mol. Cell.

[b19] Laplante M, Sabatini DM (2012). mTOR signaling in growth control and disease. Cell.

[b20] López-Maury L, Marguerat S, Bähler J (2008). Tuning gene expression to changing environments: from rapid responses to evolutionary adaptation. Nat. Rev. Genet.

[b21] Lublin A, Isoda F, Patel H, Yen K, Nguyen L, Hajje D, Schwartz M, Mobbs C (2011). FDA-approved drugs that protect mammalian neurons from glucose toxicity slow aging dependent on cbp and protect against proteotoxicity. PLoS ONE.

[b22] Lyne R, Burns G, Mata J, Penkett CJ, Rustici G, Chen D, Langford C, Vetrie D, Bähler J (2003). Whole-genome microarrays of fission yeast: characteristics, accuracy, reproducibility, and processing of array data. BMC Genomics.

[b23] Mata J, Bähler J (2006). Global roles of Ste11p, cell type, and pheromone in the control of gene expression during early sexual differentiation in fission yeast. Proc. Natl Acad. Sci. USA.

[b24] Mata J, Lyne R, Burns G, Bähler J (2002). The transcriptional program of meiosis and sporulation in fission yeast. Nat. Genet.

[b25] Matsuo T, Otsubo Y, Urano J, Tamanoi F, Yamamoto M (2007). Loss of the TOR kinase Tor2 mimics nitrogen starvation and activates the sexual development pathway in fission yeast. Mol. Cell. Biol.

[b26] Murakami CJ, Wall V, Basisty N, Kaeberlein M (2011). Composition and acidification of the culture medium influences chronological aging similarly in vineyard and laboratory yeast. PLoS ONE.

[b27] Nakashima A, Sato T, Tamanoi F (2010). Fission yeast TORC1 regulates phosphorylation of ribosomal S6 proteins in response to nutrients and its activity is inhibited by rapamycin. J. Cell Sci.

[b28] Pan Y, Shadel GS (2009). Extension of chronological life span by reduced TOR signaling requires down-regulation of Sch9p and involves increased mitochondrial OXPHOS complex density. Aging.

[b29] Petersen J, Nurse P (2007). TOR signalling regulates mitotic commitment through the stress MAP kinase pathway and the Polo and Cdc2 kinases. Nat. Cell Biol.

[b30] Powers RW, Kaeberlein M, Caldwell SD, Kennedy BK, Fields S (2006). Extension of chronological life span in yeast by decreased TOR pathway signaling. Genes Dev.

[b100] Rasband WS (1997). http://imagej.nih.gov/ij/.

[b31] Rosso A, Mossey J, Lippa CF (2008). Caffeine: neuroprotective functions in cognition and Alzheimer’s disease. Am. J. Alzheimers. Dis. Other. Demen.

[b32] Roux AE, Leroux A, Alaamery MA, Hoffman CS, Chartrand P, Ferbeyre G, Rokeach LA (2009). Pro-aging effects of glucose signaling through a G protein-coupled glucose receptor in fission yeast. PLoS Genet.

[b33] Sarkaria JN, Busby EC, Tibbetts RS, Roos P, Taya Y, Karnitz LM, Abraham RT (1999). Inhibition of ATM and ATR kinase activities by the radiosensitizing agent, caffeine. Cancer Res.

[b34] Takahara T, Maeda T (2012). TORC1 of fission yeast is rapamycin-sensitive. Genes Cells.

[b35] Turner JJ, Ewald JC, Skotheim JM (2012). Cell size control in yeast. Curr. Biol.

[b36] Urban J, Soulard A, Huber A, Lippman S, Mukhopadhyay D, Deloche O, Wanke V, Anrather D, Ammerer G, Riezman H, Broach JR, De Virgilio C, Hall MN, Loewith R (2007). Sch9 is a major target of TORC1 in *Saccharomyces cerevisiae*. Mol. Cell.

[b37] Uritani M, Hidaka H, Hotta Y, Ueno M, Ushimaru T, Toda T (2006). Fission yeast Tor2 links nitrogen signals to cell proliferation and acts downstream of the Rheb GTPase. Genes Cells.

[b38] Wang SW, Norbury C, Harris AL, Toda T (1999). Caffeine can override the S-M checkpoint in fission yeast. J. Cell Sci.

[b39] Wanke V, Cameroni E, Uotila A, Piccolis M, Urban J, Loewith R, De Virgilio C (2008). Caffeine extends yeast lifespan by targeting TORC1. Mol. Microbiol.

[b40] Wei M, Fabrizio P, Hu J, Ge H, Cheng C, Li L, Longo VD (2008). Life span extension by calorie restriction depends on Rim15 and transcription factors downstream of Ras/PKA, Tor, and Sch9. PLoS Genet.

[b41] Weisman R (2010). Fission Yeast Tor and Rapamycin. The Enzymes.

[b42] Weisman R, Finkelstein S, Choder M (2001). Rapamycin blocks sexual development in fission yeast through inhibition of the cellular function of an FKBP12 homolog. J. Biol. Chem.

[b43] Wullschleger S, Loewith R, Hall MN (2006). TOR signaling in growth and metabolism. Cell.

[b44] Yanagida M (2009). Cellular quiescence: are controlling genes conserved?. Trends Cell Biol.

[b45] Zuin A, Carmona M, Morales-Ivorra I, Gabrielli N, Vivancos AP, Ayte J, Hidalgo E (2010). Lifespan extension by calorie restriction relies on the Sty1 MAP kinase stress pathway. EMBO J.

