# Supplementary material for: TORC1 signaling inhibition by rapamycin and caffeine affect lifespan, global gene expression, and cell proliferation of fission yeast
Source: Aging Cell. 2013 May 2;12(4):563–73. doi: 10.1111/acel.12080 (PMC3798131; doi:10.1111/acel.12080)
Supplement: Fig S1 — Survival assays of wild-type cells following drug treatment in YES and EMM media showing logarithmic scales of cell survival. [file acel0012-0563-sd1.pdf]

Figure S1.

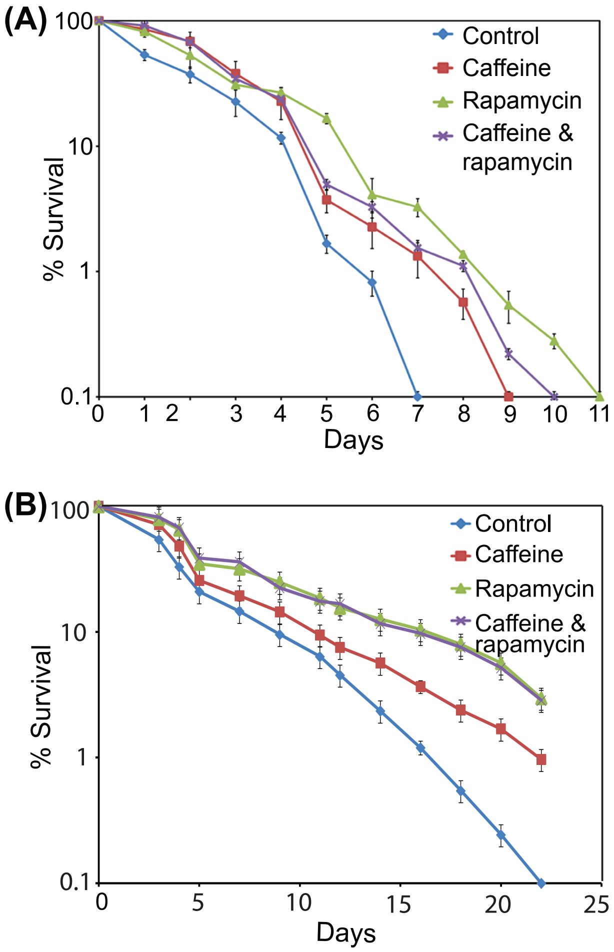

Fig.S1. Representation of CLS assays in a logarithmic scale shows differences in medial lifespans.
(A) CLS assay performed in YES media and shown in Fig.1C, here with cells survival shown in linear scale instead of logarithmic.
(B) CLS assay performed in EMM media and shown in Fig.1D here with cells survival shown in linear scale instead of logarithmic. Caffeine and rapamycin concentrations are10 mM and 100 μg/ml respectively in both panels.
