# Supplementary material for: TORC1 signaling inhibition by rapamycin and caffeine affect lifespan, global gene expression, and cell proliferation of fission yeast
Source: Aging Cell. 2013 May 2;12(4):563–73. doi: 10.1111/acel.12080 (PMC3798131; doi:10.1111/acel.12080)
Supplement: Fig S2 — Parallel measurements of cell growth and pH in media without (control) or with drugs as indicated, and CLS in buffered YES media with or without drugs as indicated [file acel0012-0563-sd2.pdf]

Figure S2.

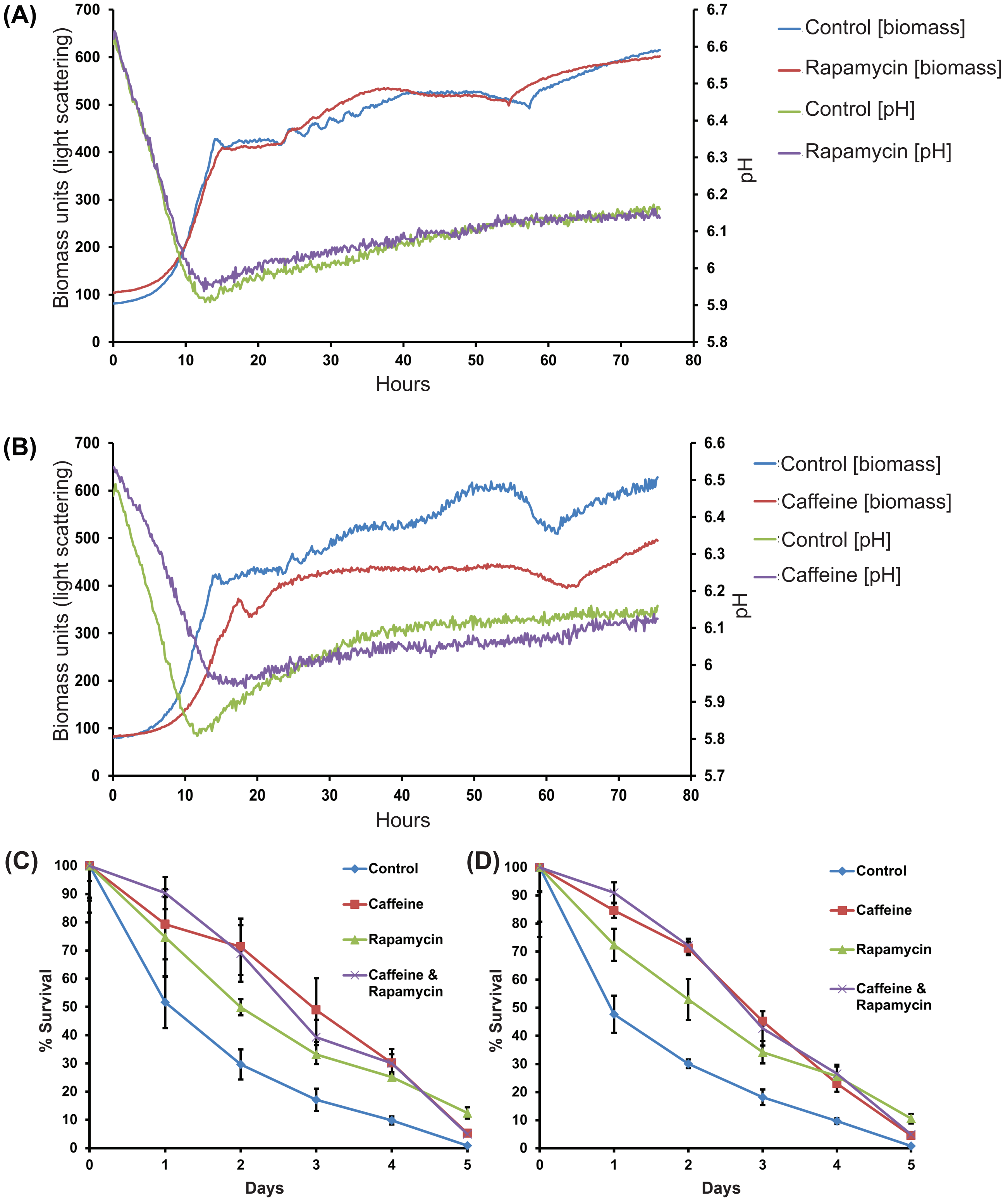

Fig. S2. Effects of rapamycin and caffeine on YES media pH during growth and stationary phase and CLS of fission yeast cells in buffered YES media with or without drug treatment.
(A) Parallel measurements of growth and pH with or without rapamycin as indicated.
(B) Parallel measurements of growth and pH with or without caffeine as indicated.
(C) CLS in buffered YES media at pH 6 with or without caffeine or rapamycin as indicated.
(D) CLS in buffered YES media at pH 4 with or without caffeine or rapamycin as indicated.
Caffeine and rapamycin are used in concentrations 10 mM and 100 μg/ml respectively.
