# Supplementary material for: TORC1 signaling inhibition by rapamycin and caffeine affect lifespan, global gene expression, and cell proliferation of fission yeast
Source: Aging Cell. 2013 May 2;12(4):563–73. doi: 10.1111/acel.12080 (PMC3798131; doi:10.1111/acel.12080)
Supplement: Fig S3 — Cell morphology and DNA content in caffeine and rapamycin-treated cells. [file acel0012-0563-sd3.pdf]

Figure S3.

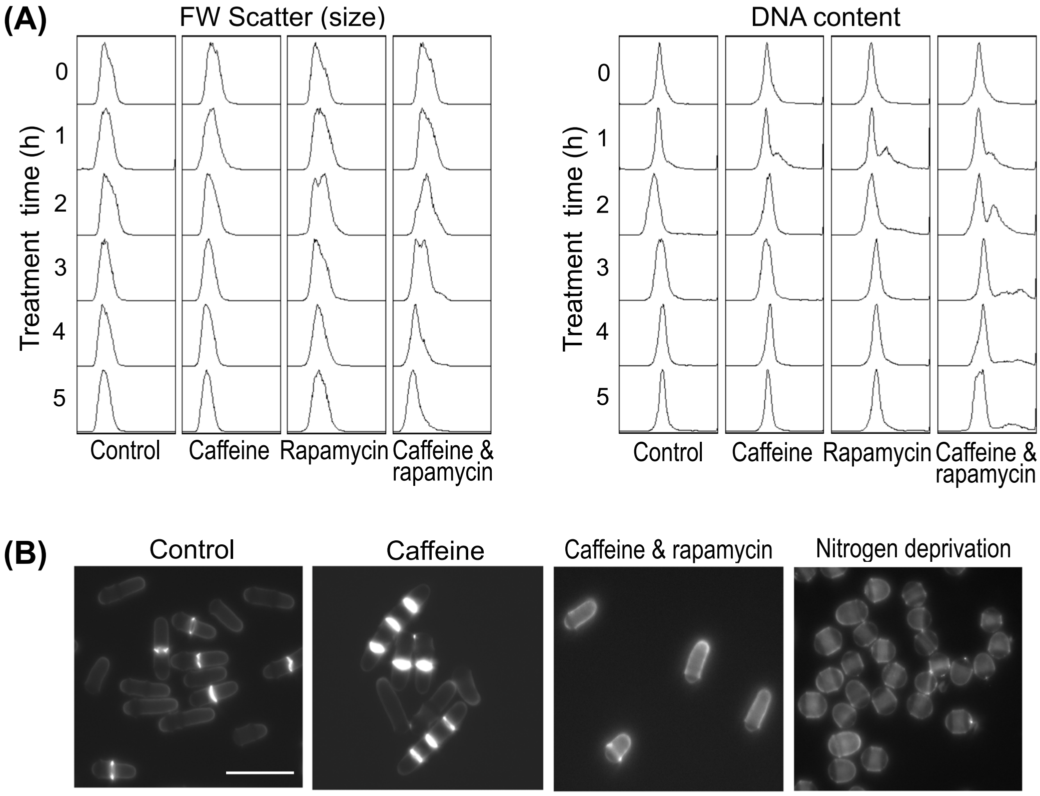

Fig. S3 Cell morphology and DNA content in caffeine and rapamycin treated cells.
(A) FACS analysis showing the changes in cell size (FW Scatter) and DNA content of cells treated with rapamycin and caffeine . Combined drug treatment leads to cell size decrease of the whole population. Caffeine treatment, singly and combined with rapamycin, leads to a portion of the cell population to exhibit a multi-septated phenotype (small peak at right).
(B) Morphology of cells after 5h of different drug treatments as indicated. Bar equals 15 μm.
Cell were grown and treated with the drugs in YES medium.
