# Supplementary material for: TORC1 signaling inhibition by rapamycin and caffeine affect lifespan, global gene expression, and cell proliferation of fission yeast
Source: Aging Cell. 2013 May 2;12(4):563–73. doi: 10.1111/acel.12080 (PMC3798131; doi:10.1111/acel.12080)
Supplement: Fig S4 — Growth analysis of cells in EMM media untreated (control) or treated with drugs as indicated. [file acel0012-0563-sd4.pdf]

Figure S4.

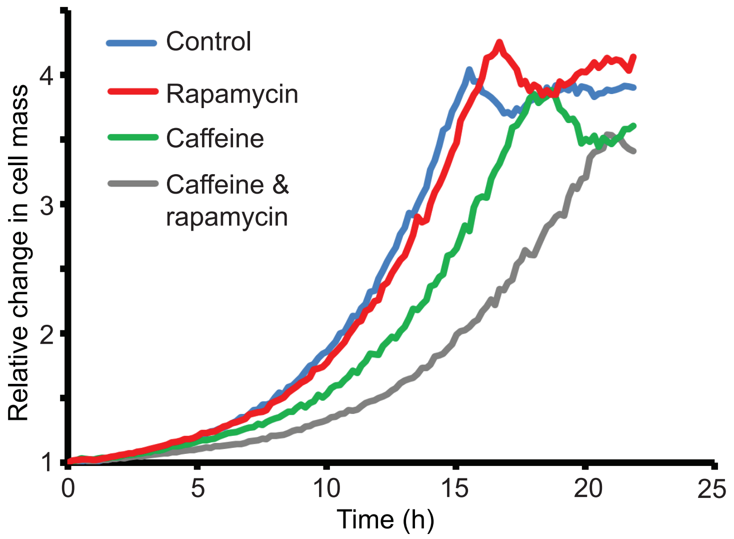

Fig. S4 Growth curves of wild-type cells in liquid EMM medium without (control) or with different drug treatments as indicated.
