# Supplementary material for: TORC1 signaling inhibition by rapamycin and caffeine affect lifespan, global gene expression, and cell proliferation of fission yeast
Source: Aging Cell. 2013 May 2;12(4):563–73. doi: 10.1111/acel.12080 (PMC3798131; doi:10.1111/acel.12080)
Supplement: Fig S5 — Correlation of cell viability with the concentrations of rapamycin and caffeine administered to growing cells. [file acel0012-0563-sd5.pdf]

Fig. S5

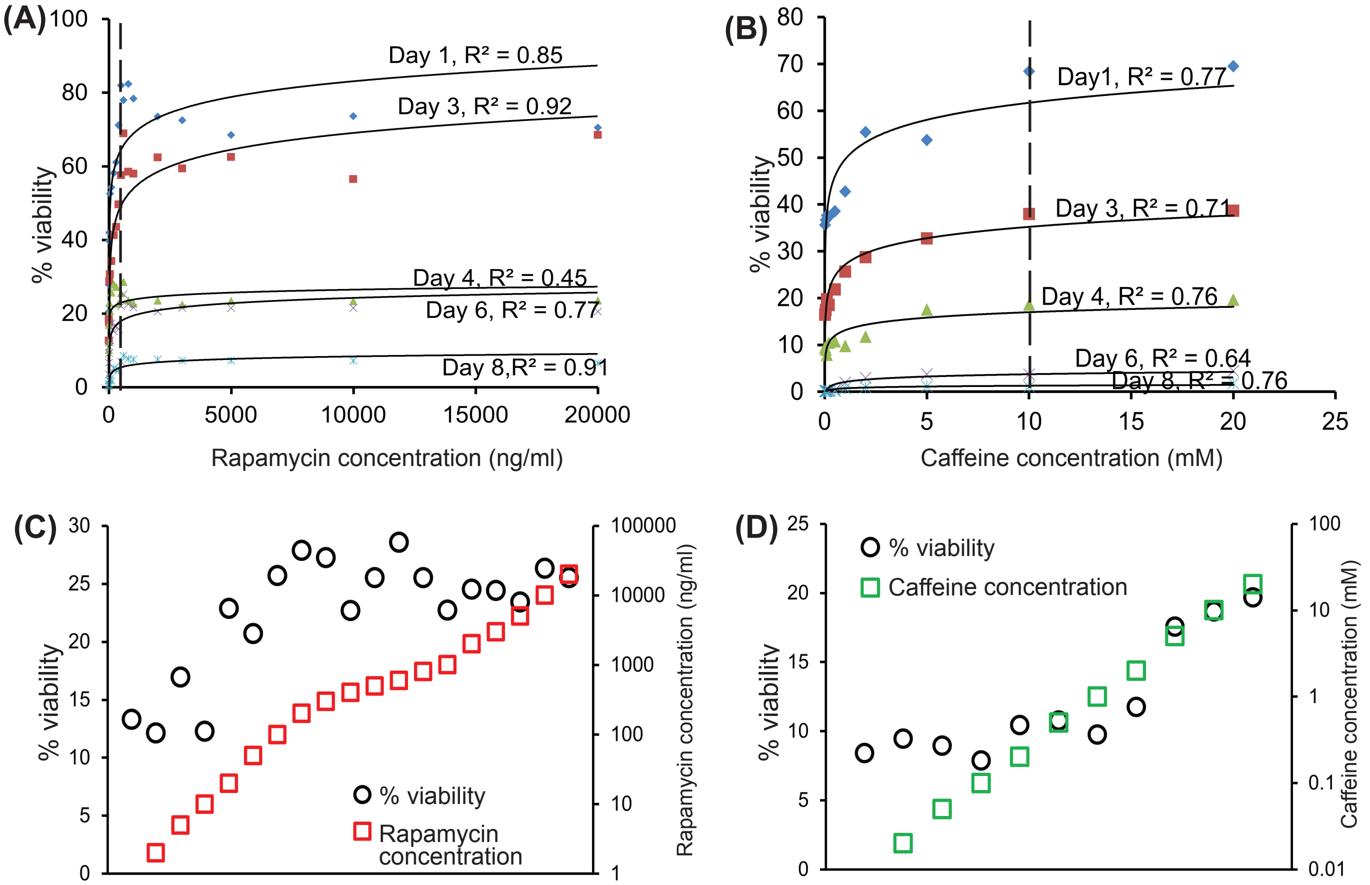

(A) Correlation of cell viability with the concentration of rapamycin administered to growing cells.
Regression model curve and R-squared (squared Pearson correlation coefficient) is indicated for the different days in stationary phase. Vertical black dashed lines indicate the standard concentration of rapamycin used in the other assays of the paper.
(B) Correlation of cell viability with the concentration of caffeine administered to growing cells.
Regression model curve and R-squared (squared Pearson correlation coefficient) is indicated for the different days in stationary phase. Vertical black dashed lines indicate the standard concentration of caffeine used in the other assays of the paper.
(C) Correlation of cell viability with the concentration of rapamycin administered to growing cells.
The graph shows data from from day 4 of CLS assay.
(D) Correlation of cell viability with the concentration of caffeine administered to growing cells.
The graph shows data from from day 4 of CLS assay.
